# The effectiveness of topical fluoride agents on preventing development of approximal caries in primary teeth: a randomized clinical trial

**DOI:** 10.1186/s12903-023-03045-4

**Published:** 2023-06-02

**Authors:** Parach Sirivichayakul, Varangkanar Jirarattanasopha, Araya Phonghanyudh, Pitchaya Tunlayadechanont, Ploychompoo Khumsub, Duangporn Duangthip

**Affiliations:** 1 Department of Pediatric Dentistry, Faculty of Dentistry, Mahidol University, No. 6, Yothi Road, Ratchathewi District, Bangkok, 10400 Thailand; 2 School of Dentistry, King Mongkut’s Institute of Technology Ladkrabang, 1 Chalongkrung St, Ladkrabang, Bangkok, 10520 Thailand; 3 Dental department, Pakkred hospital, 33 Moo 5 Saphan Nonthaburi – Bang Bua Thong Rd., Pakkret district, Nonthaburi, 11120 Thailand; 4 Faculty of Dentistry, The University of Hong Kong, Prince Phillip Dental Hospital, 34 Hospital Rd., Sai Ying Pun, Hong Kong SAR, China

**Keywords:** Early childhood caries, Enamel caries, Approximal surfaces, Primary teeth, Silver diamine fluoride, Fluoride varnish

## Abstract

**Background:**

This 18-month randomized clinical trial aimed to compare the effectiveness of two topical fluoride applications versus placebo control on preventing development of approximal caries in primary teeth.

**Methods:**

Preschool children were recruited if they had at least one initial approximal carious lesion at the distal surface of the canines, both approximal surfaces of the first molars, or the mesial surface of the second molars assessed from bitewing radiographs. The participants were randomly allocated into 3 intervention groups: Group 1 (placebo control), Group 2 (5% sodium fluoride [NaF] varnish), and Group 3 (38% silver diamine fluoride [SDF]). All agents were applied semiannually. Two calibrated examiners evaluated the caries development from bitewing radiographs. Caries development was recorded when the baseline sound surface or initial approximal carious lesion surface developed dentin caries (beyond the outer one-third of dentine) at the follow-up examination. The intention-to-treat approach was adopted. The Chi-square test was used to analyze the effectiveness of topical fluoride agents in preventing approximal caries development and the effect of other variables. The multi-level logistic regression analysis was performed to assess the relative effectiveness of topical fluoride agents in preventing approximal caries development at the 18-month follow-up.

**Results:**

At baseline, 190 participants with 2,685 sound or initial carries at the approximal surfaces were recruited. No differences in participant demographic backgrounds, oral health related habits, or caries experience were observed among the 3 groups (*P* > 0.05). After 18 months, 155 (82%) participants remained in the study. The rates of developing approximal caries in Groups 1, 2, and 3 were 24.1%, 17.1%, and 27.2%, respectively (*P* < 0.001, *χ*^*2*^ test). After adjusting for confounding factors and clustering effect, the multilevel logistic regression analysis showed no differences in caries development rates between the 3 groups (*P* > 0.05). Tooth type and the extent of a carious lesion at baseline were the significant factors for caries development.

**Conclusion:**

At 18-month follow-up, after adjusting for confounding factors and clustering effect, there were no statistically significant differences in preventing of approximal caries development between the semiannual application of 5%NaF, 38%SDF, or placebo.

**Trial registration:**

The study was registered in the Thai Clinical Trials Registry under the number TCTR20190315003 on 15/03/2019.

## Background

Early childhood caries (ECC) remains highly prevalent in many countries [1]. At an early age, the upper anterior teeth and occlusal surfaces of posterior teeth are the most affected areas. Once the primary dentition has fully erupted and the posterior teeth have formed contacts with the adjacent teeth, the approximal surfaces of posterior primary teeth become highly sensitive areas for caries formation [2]. Approximal caries in children is very common even in areas with a low caries population, one-third of 5-year-old Swedish children had one or more approximal enamel and dentine lesions [3]. This surface has a rapid rate of caries progression. It takes approximately 10 months from the initial stage to progress to the stage of radiolucency to the dentino-enamel junction and 2 years from the early stages to the inner half of dentine [4]. Routine tooth brushing is inadequate in cleaning approximal surfaces. Although dental floss is an interdental cleaning tool, limited evidence indicates that flossing can prevent dental caries [5]. Furthermore, children’s behavioral issues, limited fine motor skills, and parental reliance on health care all contribute to the development of ECC. Therefore, strategies that do not depend on individuals, such as professional fluoride applications for the control of ECC, are required to enhance the effectiveness of routine oral health care.

Sodium fluoride (NaF) 5% varnish is a professional topical fluoride recommended for application in moderate and high caries-risk children every 3–6 months to prevent caries development. The mechanism of action of fluoride is the inhibition demineralization and enhancement of remineralization [6]. The use of fluoride varnish provides a potential benefit over other forms of fluoride in terms of prolonged contact time, acting as a slow-release reservoir to prevent the rapid loss of fluoride after application [7]. It can be applied easily and quickly in young children [7]. A Cochrane systematic review reported 37% preventive fraction of fluoride varnish in primary teeth [8] and systematic reviews by Gao et al. showed that the overall percentage of remineralized enamel caries with fluoride varnish was 63.6% [9]. Fluoride varnish applied to the entire dentition is expected to prevent caries development on the approximal surfaces, just as it does on other surfaces. Evidence regarding the effectiveness of fluoride varnish was mainly based on visual examinations that persistently overlooked carious lesions on approximal surfaces, particularly initial to moderate carious lesions, which can be detected by bitewing radiographic examination. Few studies have evaluated the effectiveness of fluoride varnish on approximal surfaces under radiographic examination in primary dentition [10–12].

Silver diamine fluoride (SDF) is another popular high concentration topical fluoride that has remarkable effectiveness in treating dentine caries [9, 13]. Some research investigated the caries-preventive effect of 38% SDF, which showed promising results to help prevent caries development in both primary and permanent dentition [14–16]. Furthermore, SDF appeared to provide more successful protection than NaF varnish. A pilot study on occlusal surfaces of permanent molars reported the effectiveness of SDF in controlling incipient enamel caries [17]. A randomized controlled trial showed comparable results of 30% SDF and 5% NaF varnish in controlling the progression of cavitated enamel caries [18] without placebo control. Further studies are needed to warrant or refute the preventive effect of SDF on approximal caries development compared to a control.

Both topical fluoride agents are child friendly interventions; however, a limited number of randomized clinical trials have evaluated the caries-preventive effects on approximal surfaces of primary teeth. Therefore, this study aimed to evaluate the effectiveness of two professional fluoride agents on preventing approximal carious development in primary teeth in high caries-risk preschool children: 5% NaF varnish and 38% SDF. The null hypothesis was that the approximal caries development rates of the group either treated with 5% NaF varnish or 38% SDF were comparable to that of the group treated with placebo.

## Methods

The study design was a three-arm parallel group, randomized clinical trial that allocated subjects into a 1:1:1 ratio. The trial was approved by the Institutional Review Board of the Faculty of Dentistry/Faculty of Pharmacy, Mahidol University (No. MU-DT/PY-IRB 2019/005.1101) and registered in the Thai Clinical Trials Registry (No. TCTR20190315003) on 15/03/2019.

The research purposes, procedures, risks, and benefits of the study were explained to the parents or guardians of the participants before they signed informed consent forms. All participants were free to see a dentist and had rights to withdraw from the study for any reason at any time during the trial. Informed consent was obtained from the parent or guardian of each child prior to commencing the intervention. Participant recruitment and data collection were conducted from March 2019 to October 2020.

### Participants

Children in six public schools in Nonthaburi Province located at the central region of Thailand were invited to participate in the study. The fluoride concentration in the drinking water in the study area was less than 0.03 ppm. The inclusion criteria were healthy children aged 4–6 years who could cooperate with the clinical examination and had at least one quadrant showing sound contact surfaces of posterior teeth. Children were excluded if they had a history of fluoride, silver, or colophony agent allergy, or had undergone topical fluoride administration within the previous six months or were resistant to bitewing radiography.

The inclusion criteria at the tooth surface level were the distal surfaces of the canine or first molar, or the mesial surfaces of the first or second molars showing clinically sound and radiographically sound or initial carious lesion. Initial approximal carious lesions were defined as radiolucency confined to the enamel or outer third of dentine (RA1–RA3 according to the International Caries Detection and Assessment System/International Caries Classification and Management System (ICDAS/ICCMS™) radiographic scoring system) [19].

### Interventions

Before the examination, all participants practiced tooth-brushing with our research team, were given dietary advice, and received a set of oral health care packages which included toothbrushes, fluoride toothpaste, dental floss, and leaflets on oral health education.

The participants were randomly assigned to one of the three intervention groups: Group 1 (placebo control; water), Group 2 (5% NaF varnish [Duraphat, Colgate Palmolive, USA]), and Group 3 (38% SDF [Topamine, Dentalife, Australia]) using the stratified block randomization approach with a block size of six.

All clinical procedures were performed at the participants’ schools by two dentists who were trained and calibrated to apply NaF varnish and SDF by pediatric dentist (V.J) prior to trial. The amount of apply NaF varnish applied per child was no more than 0.25 ml, and the amount of SDF applied per child was no more than 1 drop (25 µL).

Before applying either placebo or topical fluoride, the teeth were cleaned with gauze and dental floss. A disposable micro-applicator was used to apply one of the topical fluoride treatments or placebo to all tooth surfaces, paying special attention to the proximal surfaces of posterior teeth and avoiding the pulp-involved carious surfaces. After intervention, the participants were instructed to refrain from eating or drinking for at least 30 min and were monitored by their class teachers. The interventions were performed every 6 months.

### Outcome assessment

The outcome of this study was the presence of approximal carious lesions of the baseline recruited tooth surface reaching beyond the outer half of dentine after application of the intervention agents at the 6-, 12-, and 18-month follow-ups based on radiograph examinations.

### Sample size

The sample size was calculated based on data from a previous study of approximal caries development that was approximately 20.7% in a 2-year follow-up of children < 7 years old [20]. A clinically significant difference between the intervention groups would be 10%. The power of this study was set at 80% with α = 0.05 as the statistical significance level. Therefore, the calculated sample size was 219 tooth surfaces per group or 657 in total at baseline. Following the estimated intraclass correlation coefficient of 0.13 [21] and the average of sound or initial proximal tooth surfaces in each child at 12 at baseline, the design effect was 2.43. Therefore, a total of 135 children with 1600 approximal tooth surfaces would be required. Anticipating a 20% drop-out rate, a total of 168 children (at least 56 children per group) with 2012 tooth surfaces were needed to be recruited at baseline.

### Randomization and blinding

One of the 3 intervention groups was assigned to each participant. Based on stratified block randomization of six, an Excel program generated the randomization assignment. An assistant who was not involved in the study handled the participant allocation assignments in sealed envelopes and arranged them sequentially. Each envelope was opened right before the intervention was performed. The operators were not blinded to the intervention groups, but all participants, parents, and the evaluators were blinded.

### Data collection

The baseline data on the child’s demographics (age and gender), family’s socioeconomic background (parents’ education level and family incomes), and child’s oral health related behaviors (tooth brushing, use of fluoride toothpaste, and cariogenic snack consumption) were collected from the parents using a structured questionnaire.

Clinical examinations at baseline and follow-up visits were performed by a single examiner (P.S) trained and calibrated by dental specialists in dental public health (D.D) and pediatric dentistry (V.J) until the weighted kappa values of inter- and intra-reliability were ≥ 0.8 each. The examiner was blinded to the allocation groups. The clinical examinations were conducted in the school-based setting using World Health Organization (WHO) CPI periodontal probes with an LED dental mouth mirror. The participants were examined in the supine position on a mobile dental unit. The tooth/surface status was recorded as sound, decayed, missing, and filled (dmft/dmfs) based on WHO criteria. The oral cleanliness status was assessed using the simplified oral hygiene index on the buccal or lingual surfaces of 6 index teeth (55, 51, 65, 71, 75, and 85). The plaque score was measured on a scale of 0–3. An average high score indicated poor dental hygiene. The presence of food impaction and contact area characteristics (open or closed) of each proximal surface were also recorded.

Bitewing radiographic images were taken on the posterior teeth with closed contact surfaces, at the initial examination and at the 6-, 12-, and 18-month follow-ups. An experienced radiology technician conducted the radiographic imaging using size 0 digital film, a film holder, and a portable digital x-ray machine (EzRay Air: VEX-P300; 65 kV and 2.5 mA, exposure time 0.38s) with a standardized technique. Bite position was recorded and used for each follow-up visit. Both technician and participants wore lead aprons and thyroid shields while taking the radiographs. The digital films were processed automatically (VistaScan Mini Easy, Dürr Dental, Germany). The bitewing radiographic images were sent anonymously to two independent dentists trained and calibrated by a pediatric dental specialist (V.J) until inter- and intra-reliability were reached (≥ 0.8 for each dentist). The two examiners viewed the radiographic images independently via DBSWIN imaging software (Dürr Dental, Germany) and scored approximal surface characteristics accordingly: (sound) no radiolucency, (RA1) radiolucency confined to the outer half of enamel, (RA2) radiolucency that reached the inner half of the enamel or to the enamel-dentin junction, (RA3) radiolucency that reached the outer third of the dentin, (RB4) radiolucency that reached the middle third of dentin, (RC5) radiolucency that reached the inner third of dentin, (RC6) radiolucency approaching the pulp, (F) filled surface, and (E) extracted tooth. The examiners were requested to re-evaluate the surfaces with discrepancy scores. If disagreement persisted, a discussion was held until agreement was reached. Caries development was determined when the recruited approximal surface had a carious lesion at the moderate stage (RB4) or extensive stages (RC5, RC6, F, E) at the follow-up visits.

After examination, the parents/guardians of all participants received a summary of their children’s oral health status and treatment needs, leaflets of oral health education, as well as a referral letter for preventive and operative treatment.

### Statistical analysis

Data were analyzed using SPSS version 26.0 for Windows (SPSS Inc., Chicago, IL, USA). The level of statistical significance was set at *P* < 0.05. Chi-square test was used to compare baseline sociodemographic backgrounds, oral health-related behaviors of the participants, baseline approximal caries statuses, and proximal surface characteristics among the control, 38% SDF, and 5% NaF varnish groups. The comparability of the participants’ age, plaque index, and dmft/dmfs scores at baseline among the groups was assessed using analysis of variance or the Kruskal-Wallis test depending on the normality of distribution.

An intention-to-treat analysis was performed. The carious lesion status of the drop-out participants or participants who received restorative treatment or extraction between follow-up visits were recorded as caries development. The Chi-square test was used to analyze the effectiveness of topical fluoride agents in preventing approximal caries development and the association of other variables on approximal caries development. Since more than one approximal surfaces were chosen from one tooth and one child, the multi-level logistic regression analysis was performed to assess the relative effectiveness of topical fluoride agents in preventing approximal caries development at the 18-month follow-up. Effects of potential variables, including baseline demographic background, oral health-related habits, baseline clinical characteristics, on approximal caries development were also evaluated by this analysis.

## Results

**Fig. 1:**
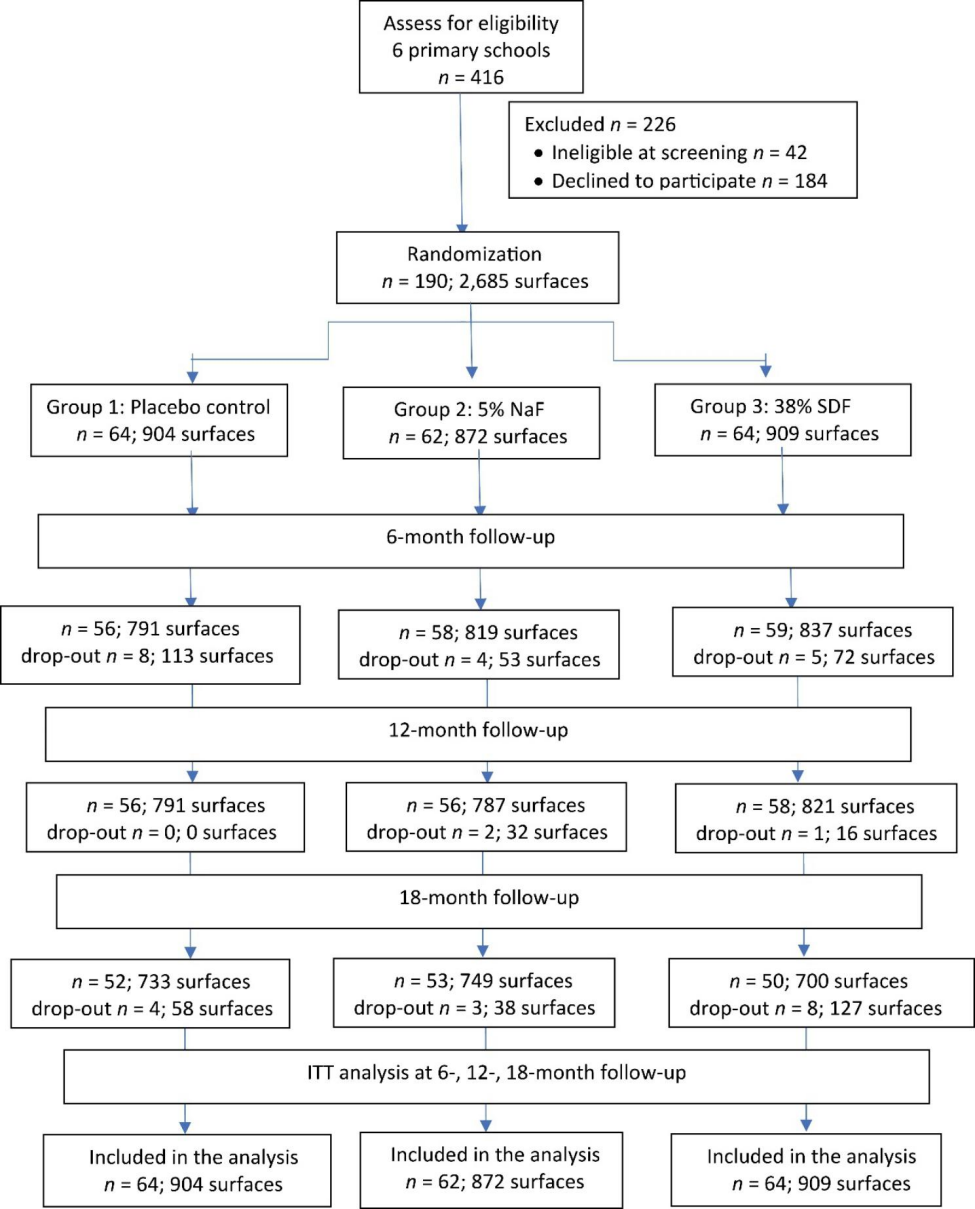
Flow chart of participants over the 18-month study period

This study enrolled 190 children (95 males and 95 girls) with an average (standard deviation [SD]) age of 60 (10) months. The number of children in Group 1 (control group), Group 2 (semi-annual NaF varnish application), and Group 3 (semi-annual SDF application) were 64, 62, and 64, respectively (Fig. 1). Children in Groups 1, 2, and 3 had 698 (77.2%), 684 (78.4%), and 673 (74.0%) sound approximal surfaces and 206 (22.8%), 188 (21.6%), 236 (26%), approximal surfaces with initial caries, respectively. There were no differences in the distribution of caries characteristics among the 3 groups (*P* = 0.326) (Table 1). The overall mean (SD) dmft and dmfs scores were 5.3 (4.3) and 11.2 (11.7), respectively. More than two thirds of the children brushed their teeth without supervision. Their demographic background, oral health related habits, and clinical characteristics were balanced across the 3 groups (*P* > 0.05) (Table 1).

**Table 1:** Demographic Background, Oral Health-related Habits, and Clinical Characteristics of Participants at Baseline

Demographic background	Group 1 Placebo control *n* = 64	Group 2 5% NaF *n* = 62	Group 3 38% SDF *n* = 64	*P* Value
Age, mean (SD), mo.	61.4 (9.8)	60.1 (10.8)	58.7 (10.6)	0.345^a^
min-max, mo.	47–81	43–81	40–83	
Gender				
male	33 (51.6)	28 (45.2)	34 (53.1)	0.640
female	31 (48.4)	34 (54.8)	30 (46.9)	
Main caregiver				
parents	49 (76.6)	46 (74.2)	51 (79.7)	0.764
others	15 (23.4)	16 (25.8)	13 (20.3)	
Father education level				
primary school	14 (21.9)	16 (25.8)	14 (21.9)	0.641
secondary school	42 (65.6)	35 (56.5)	36 (56.2)	
college or university	8 (12.5)	11 (17.7)	14 (21.9)	
Mother education level				
primary school	9 (14.1)	11 (17.7)	9 (14.1)	0.958
secondary school	43 (67.2)	39 (62.9)	41 (64.1)	
college and university	12 (18.8)	12 (19.4)	14 (21.9)	
Monthly family income, Thai baht				
< 10,000	11 (17.2)	11 (17.7)	16 (25.0)	0.510
10,000–20,000	36 (56.3)	39 (62.9)	31 (48.4)	
> 20,000	17 (26.6)	12 (19.4)	17 (26.6)	
Oral health related habits				
Tooth brushing by				0.704
child	54 (84.4)	49 (79.0)	51 (79.7)	
parents	10 (15.6)	13 (21.0)	13 (20.3)	
Frequency of brushing				0.421
< 2 times	12 (18.8)	8 (12.9)	7 (10.9)	
≥ 2 times	52 (81.3)	54 (87.1)	57 (89.1)	
Toothpaste				0.646
fluoride toothpaste	61 (95.3)	57 (91.9)	61 (95.3)	
non-fluoride toothpaste	3 (4.7)	5 (8.1)	3 (4.7)	
Milk				0.956
non-sugary	21 (32.8)	19 (30.6)	21 (32.8)	
sugary	43 (67.2)	43 (69.4)	43 (67.2)	
On bottle feeding				0.823
no	56 (87.5)	52 (83.9)	54 (84.4)	
yes	8 (12.5)	10 (16.1)	10 (15.6)	
Daily snack consumption				0.149
≤ 2 times	36 (56.3)	45 (72.6)	39 (60.9)	
> 2 times	28 (43.8)	17 (27.9)	25 (39.1)	
Clinical characteristics				
dmft, mean (SD)	5.1 (4.0)	5.4 (4.8)	5.5 (4.2)	0.877 ^b^
dmfs, mean (SD)	10.9 (10.5)	11.4 (12.6)	11.3 (12.1)	0.948 ^b^
plaque index, mean (SD)	1.5 (0.4)	1.5 (0.4)	1.6 (0.4)	0.398 ^a^
Carious surface characteristics	*n* = 904	*n* = 872	*n* = 909	
Tooth type				0.986
second molar	226 (25.0)	219 (25.1)	224 (24.6)	
first molar	432 (47.8)	424 (48.6)	436 (48.0)	
canine	246 (27.2)	229 (26.3)	249 (27.4)	
Tooth position				0.995
upper	451 (49.9)	437 (50.1)	455 (50.1)	
lower	453 (50.1)	435 (49.9)	454 (49.9)	
Surface type				0.978
mesial	457 (50.6)	439 (50.3)	455 (50.1)	
distal	447 (49.4)	433 (49.7)	454 (49.9)	
Extent of caries in radiograph				0.326
Sound	698 (77.2)	684 (78.4)	673 (74.0)	
RA1	124 (13.7)	108 (12.4)	129 (14.2)	
RA2	41 (4.5)	37 (4.2)	49 (5.4)	
RA3	41 (4.5)	43 (4.9)	58 (6.4)	
Contact characteristics				0.274
closed	633 (70.0)	595 (68.2)	652 (71.7)	
open	271 (30.0)	277 (31.8)	257 (28.3)	
Food impaction				0.599
yes	47 (5.2)	54 (6.2)	48 (5.3)	
no	857 (94.8)	818 (93.8)	861 (94.7)	
Adjacent surface				0.264
sound	686 (75.9)	663 (76.0)	655 (72.1)	
non-cavitated caries	185 (20.5)	175 (20.1)	218 (24.0)	
cavitated caries	33 (3.7)	34 (3.9)	36 (4.0)	

^a^ ANOVA

^b^ Kruskal-Wallis test

Data are presented as *n* (%) unless otherwise indicated

At the 18-month follow-up, 155 children (82.6%) remained in the trial. There were no significant differences between the subject dropout rates among three groups (18.8%, 14.5%, and 21.9% in Groups 1, 2, and 3, respectively with *P* = 0.565). All (100%) children who dropped out from the study were due to changing the school (not related to the study).

Table 2 shows the caries development rates in the NaF varnish and SDF groups compared to the control group at the 6-, 12-, and 18-month follow-ups. The caries development rates increased over time regardless of the intervention group. Furthermore, the caries development rates for the baseline sound approximal surface were statistically significantly different among the 3 groups at all follow-up visits. No significant differences were detected in the caries development rates among the groups for the baseline approximal surface with initial carious lesion. However, the overall approximal caries development rates of Groups 1, 2, and 3 at the 18-month follow-up were 24.1%, 17.1%, and 27.2%, respectively, which were statistically significantly different among the 3 groups at each follow-up (*P* < 0.05). Group 2 had the lowest incidence of caries development.

**Table 2:** Caries Development Rate at the 6-, 12-, and 18-month Follow-ups Among 3 Groups

	Group 1 Control	Group 2 5% NaF	Group 3 38% SDF	*P* Value^a^
Overall	*n* = 904	*n* = 872	*n* = 909	
6 months	131 (14.5)	61 (7.0)	88 (9.7)	< 0.001
12 months	151 (16.7)	107 (12.3)	115 (12.7)	0.011
18 months	218 (24.1)	149 (17.1)	247 (27.2)	< 0.001
Sound surface	*n* = 698	*n* = 684	*n* = 673	
6 months	94 (13.5)	37 (5.4)	54 (8.0)	< 0.001
12 months	96 (13.8)	61 (8.9)	59 (8.8)	0.003
18 months	142 (20.3)	87 (12.7)	151 (22.4)	< 0.001
Initial caries (RA1-2-3)	*n = 206*	*n* = 188	*n* = 236	
6 months	37 (18.0)	24 (12.8)	34 (14.4)	0.332
12 months	55 (26.7)	46 (24.5)	56 (23.7)	0.760
18 months	76 (36.9)	62 (33.0)	96 (40.7)	0.264

Data are presented as *n* (%)

^a^Chi-square test

The associations between caries development rates and other variables, such as demographic background, oral health-related behavior, and clinical features, are shown in Table 3. Several potential factors could influence the rate of caries development. Therefore, multi-level logistic regression analyses were performed at the 18-month follow-up (Table 4). After adjusting for clustering effect and confounding factors, the multi-level logistic regression analysis showed no differences in caries development among the 3 groups (*P* > 0.05). Tooth type and the extent of caries lesion at baseline were significant factors for caries development (*P* < 0.05).

**Table 3:** Association Between Caries Development Rate with Independent Variables at 18 Months Follow-up: Univariate analysis (*n* = 2685 surfaces)

	Caries development rate *n* (%)	*P* Value
Gender		0.496
male	302 (22.3)	
female	312 (23.4)	
Main caregiver		0.006
parents	494 (24.1)	
others	120 (18.9)	
Father education level		< 0.001
primary	115 (18.8)	
secondary	413 (25.7)	
college	86 (18.4)	
Mother education level		0.076
primary	77 (18.7)	
secondary	405 (23.3)	
college	132 (24.5)	
Monthly family income, Thai baht		0.630
< 10,000	126 (23.6)	
10,000–20,000	332 (22.2)	
> 20,000	156 (23.9)	
Tooth brushing by		0.003
child	518 (24.1)	
parents	96 (18.0)	
Frequency of brushing		< 0.001
< 2 times	113 (32.0)	
≥ 2 times	501 (21.5)	
Brushing with fluoride toothpaste		0.018
yes	570 (22.4)	
no	44 (31.0)	
Milk		< 0.001
non-sugary	156 (18.0)	
sugary	458 (25.2)	
On bottle feeding		0.954
yes	91 (23.0)	
no	523 (22.8)	
Daily snack consumption		0.127
≤ 2 times	373 (21.9)	
> 2 times	241 (24.5)	
Contact characteristics		0.056
closed	449 (23.9)	
open	165 (20.5)	
Food impaction		< 0.001
yes	52 (34.9)	
no	562 (22.2)	
Adjacent surface characteristics		< 0.001
sound	406 (20.3)	
non-cavitated	181 (31.3)	
cavitated	27 (26.2)	

**Table 4:** Final Multi-level Logistic Regression Model of the Caries Development Rate at 18-month Follow-up

Variables	Adjusted odds ratio^a, b^	95% CI	*P* Value
Group			0.371
38% SDF	1.12	0.52–2.42	0.774
5% NaF	0.63	0.28–1.44	0.273
Placebo control	1		-
Tooth type			0.005
Second molar	1.11	0.93–1.32	0.231
First molar	1.20	1.07–1.36	0.003
Canine	1		
Baseline x-ray			< 0.001
RA3	6.82	4.15–11.19	< 0.001
RA2	3.02	1.79–5.08	< 0.001
RA1	1.50	1.01–2.21	0.043
Sound	1		

^a^ excluded non-significant variables: baseline demographic variables (child’s gender, main caregiver, education level of parents, and monthly family income), oral health-related habits (brushing habit, use of fluoride tooth paste, drinking sugary milk, bottle feeding habit, and frequency of snack taking), and baseline clinical characteristics (dmfs, plaque index, tooth surface type, presence of food impaction at the approximal area and contact characteristics). ^b^ Adjusted odds ratio > 1 corresponds to a higher chance of approximal caries development

## Discussion

A PubMed search on 3th December 2022 found no studies on the effectiveness of SDF to prevent approximal caries in primary teeth. This is the first randomized clinical trial to compare the effectiveness of 38% SDF to 5%NaF varnish on approximal surfaces with a control. The results of our study demonstrated no significant differences in preventing approximal caries development among the 3 groups after controlling for cluster effects and confounding factors based on the 18-month follow-up. The results of the present study support a recent systematic review that concluded that fluoride varnish had a modest and uncertain effect on preventing caries development in children [22, 23]. The findings of this study were also consistent with a recent systematic review and network meta-analysis [24], that for the application of 5% NaF varnish, there may be a higher chance of arresting non-cavitated lesions as compared with no treatment; however, the results were not statistically significant.

The effectiveness of semi-annual 38% SDF application on preventing caries development was also not observed in our study. Preventing caries development on approximal areas might be more difficult than other surfaces because these areas are more vulnerable to plaque accumulation. Furthermore, approximal areas are difficult to clean and they have restricted salivary access and less fluoride toothpaste exposure compared to other surfaces. The results of the current study were inconsistent with previous studies [14, 15]. The possible explanation may be the differences in examinations, such as no radiographic examination, differences in child populations with different caries risks, and also different eligibility criteria at the tooth surface level included in each study. In children with high caries risk, semi-annual applications of topical fluorides seem insufficient in preventing caries development. A more frequent application of more than twice a year as a complement to a comprehensive oral health promotion program has been recommended [25, 26]; however, the evidence supporting this recommendation has been graded as of low to very low certainty. Future studies are required to warrant or refute the efficacy of topical fluorides to prevent approximal caries using more frequent applications in high caries-risk children.

Another possibility of the topical fluoride effectiveness being not significantly different from the control group could be the Hawthorne effect [27, 28]. Since all participants were aware they would be examined on a regular basis, they all established more favorable behavioral habits. Parents in this study were also aware they were taking part in a clinical trial for the prevention of dental caries. Furthermore, the oral health status of the children was reported to their parents at each examination visit, and the importance of oral health care was emphasized to them as well, which likely led the parents to pay more attention to the oral health of their children.

The results of the multi-level logistic regression analysis demonstrated that tooth type and the extent of baseline approximal caries influenced approximal caries development. The first primary molar was more prone to caries development than the canine or second molar in this age group, which was consistent with previous studies [29, 30]. This was likely because the enamel thickness of the first primary molar is thinner than the second primary molar. In addition, the extent of baseline approximal caries was another crucial factor on caries development [31]. Our data revealed that enamel proximal caries had a higher chance to progress into cavitated dentine caries, compared to sound proximal tooth surface. It was underscored that once enamel caries is developed in the approximal surface, it is likely to progress into to the moderate stage regardless of subject characteristics or oral health related behavior. Secondary and tertiary prevention efforts are required to reduce the negative impacts of dental caries once established.

The strengths of this study include sufficient sample size, comprehensive assessment with both clinical and radiographic examinations, as well as using the ICCMS™ scoring system to compare our results to other studies in the future. This study also has limitations. The study was conducted in primary teeth of preschool children with high caries risk in a school-based setting. The results cannot be generalized to other age groups with different caries risks. More research is required regarding the effectiveness of different frequency of professionally applied topical fluorides in preventing and controlling approximal carious lesions.

As early childhood caries is a multifactorial disease, preventing new caries development or arresting the progression of carious lesions cannot be accomplished with a single intervention, such as the application of professional topical fluoride, especially in children at high risk for caries. The strategies for preventing ECC in high-risk groups require a combination of individual- and community-level interventions including hand-on tooth brushing with fluoride toothpaste, educating parents / caregivers to improve oral health literacy, promoting school and community to create a healthy dietary environment, and implementing water or milk fluoridation.

### Conclusion

Based on the 18-month results, the caries development rates increased over time regardless of the intervention group. After adjusting for clustering and confounding, no statistically significant differences in preventing approximal caries development in primary teeth were observed between the semi-annual application of 5%NaF varnish, 38%SDF, and placebo in high caries-risk preschool children in the school-based setting.

## Data Availability

The datasets generated and/or analyzed during the current study are not publicly available due to privacy and ethical concern but are available from the corresponding author on reasonable request.

## Abbreviations

ECC: Early childhood caries
NaF: Sodium fluoride
SDF: Silver diamine fluoride
ICDAS/ICCMS: International Caries Detection and Assessment System/International Caries Classification and Management System
WHO: World Health Organixation
dmft: decayed, missing and filled teeth
dmfs: decayed, missing and filled surfaces
SD: Standard deviation
CI: Confidence interval

## References

[1] Tinanoff N, Baez RJ, Diaz Guillory C, Donly KJ, Feldens CA, McGrath C (2019). Early childhood caries epidemiology, aetiology, risk assessment, societal burden, management, education, and policy: global perspective. Int J Paediatr Dent.

[2] Allison PJ, Schwartz S (2003). Interproximal contact points and proximal caries in posterior primary teeth. Pediatr Dent.

[3] Anderson M, Stecksen-Blicks C, Stenlund H, Ranggard L, Tsilingaridis G, Mejare I (2005). Detection of approximal caries in 5-year-old swedish children. Caries Res.

[4] Tickotsky N, Petel R, Araki R, Moskovitz M (2017). Caries progression rate in primary teeth: a retrospective study. J Clin Pediatr Dent.

[5] de Oliveira KMH, Nemezio MA, Romualdo PC, da Silva RAB, de Paula E, Silva FWG, Kuchler EC (2017). Dental flossing and proximal caries in the primary dentition: a systematic review. Oral Health Prev Dent.

[6] Buzalaf MAR, Pessan JP, Honório HM, Ten Cate JM (2011). Mechanisms of action of fluoride for caries control. Monogr Oral Sci.

[7] Chu CH, Lo EC (2006). A review of sodium fluoride varnish. Gen Dent.

[8] Marinho VC, Worthington HV, Walsh T, Clarkson JE. Fluoride varnishes for preventing dental caries in children and adolescents. Cochrane Database Syst Rev. 2013;CD002279. 10.1002/14651858.CD002279.pub2 10.1002/14651858.CD002279.pub2 PMC10758998 23846772

[9] Gao SS, Zhang S, Mei ML, Lo EC, Chu CH (2016). Caries remineralisation and arresting effect in children by professionally applied fluoride treatment - a systematic review. BMC Oral Health.

[10] Murray JJ, Majid ZA (1978). The prevalence and progression of approximal caries in the deciduous dentition in british children. Br Dent J.

[11] Peyron M, Matsson L, Birkhed D (1992). Progression of approximal caries in primary molars and the effect of Duraphat treatment. Scand J Dent Res.

[12] Ekstrand KR, Bakhshandeh A, Martignon S (2010). Treatment of proximal superficial caries lesions on primary molar teeth with resin infiltration and fluoride varnish versus fluoride varnish only: efficacy after 1 year. Caries Res.

[13] Seifo N, Cassie H, Radford JR, Innes NPT (2019). Silver diamine fluoride for managing carious lesions: an umbrella review. BMC Oral Health.

[14] Chu CH, Lo EC, Lin HC (2002). Effectiveness of silver diamine fluoride and sodium fluoride varnish in arresting dentin caries in chinese pre-school children. J Dent Res.

[15] Llodra JC, Rodriguez A, Ferrer B, Menardia V, Ramos T, Morato M (2005). Efficacy of silver diamine fluoride for caries reduction in primary teeth and first permanent molars of schoolchildren: 36-month clinical trial. J Dent Res.

[16] Oliveira BH, Rajendra A, Veitz-Keenan A, Niederman R (2019). The effect of silver diamine fluoride in preventing caries in the primary dentition: a systematic review and meta-analysis. Caries Res.

[17] Braga MM, Mendes FM, De Benedetto MS, Imparato JC (2009). Effect of silver diammine fluoride on incipient caries lesions in erupting permanent first molars: a pilot study. J Dent Child (Chic).

[18] Duangthip D, Wong MCM, Chu CH, Lo ECM (2018). Caries arrest by topical fluorides in preschool children: 30-month results. J Dent.

[19] Pitts NB, Ismail AI, Martignon S, Ekstrand K, Douglas GVA, Longbottom C et al. ICCMS^™^ Guide for practitioners and educators. 2014; ICDAS Foundation. https://www.iccms-web.com/uploads/asset/59284654c0a6f822230100.pdf

[20] Dean JA, Barton DH, Vahedi I, Hatcher EA (1997). Progression of interproximal caries in the primary dentition. J Clin Pediatr Dent.

[21] Fung MHT, Duangthip D, Wong MCM, Lo ECM, Chu CH (2016). Arresting dentine caries with different concentration and periodicity of silver diamine fluoride. JDR Clin Trans Res.

[22] de Sousa FSO, Dos Santos APP, Nadanovsky P, Hujoel P, Cunha-Cruz J, de Oliveira BH (2019). Fluoride varnish and dental caries in preschoolers: a systematic review and meta-analysis. Caries Res.

[23] Yu L, Yu X, Li Y, Yang F, Hong J, Qin D, Song G, Hua F (2021). The additional benefit of professional fluoride application for children as an adjunct to regular fluoride toothpaste: a systematic review and meta-analysis. Clin Oral Investig.

[24] Urquhart O, Tampi MP, Pilcher L, Slayton RL, Araujo MWB, Fontana M (2019). Nonrestorative treatments for caries: systematic review and network meta-analysis. J Dent Res.

[25] Toumba KJ, Twetman S, Splieth C, Parnell C, van Loveren C, Lygidakis N (2019). Guidelines on the use of fluoride for caries prevention in children: an updated EAPD policy document. Eur Arch Paediatr Dent.

[26] Slayton RL, Urquhart O, Araujo MWB, Fontana M, Guzmán-Armstrong S, Nascimento MM (2018). Evidence-based clinical practice guideline on nonrestorative treatments for carious lesions: a report from the american Dental Association. J Am Dent Assoc.

[27] McCarney R, Warner J, Iliffe S, van Haselen R, Griffin M, Fisher P (2007). The hawthorne effect: a randomised, controlled trial. BMC Med Res Methodol.

[28] McCambridge J, Witton J, Elbourne DR (2014). Systematic review of the hawthorne effect: new concepts are needed to study research participation effects. J Clin Epidemiol.

[29] Elfrink ME, Veerkamp JS, Kalsbeek H (2006). Caries pattern in primary molars in dutch 5-year-old children. Eur Arch Paediatr Dent.

[30] Ferro R, Besostri A, Olivieri A (2009). Caries prevalence and tooth surface distribution in a group of 5-year-old italian children. Eur Arch Paediatr Dent.

[31] Mahoney P (2010). Two-dimensional patterns of human enamel thickness on deciduous (dm1, dm2) and permanent first (M1) mandibular molars. Arch Oral Biol.

